# A Pilot Study Identifying a Set of microRNAs As Precise Diagnostic Biomarkers of Acute Kidney Injury

**DOI:** 10.1371/journal.pone.0127175

**Published:** 2015-06-16

**Authors:** Elia Aguado-Fraile, Edurne Ramos, Elisa Conde, Macarena Rodríguez, Laura Martín-Gómez, Aurora Lietor, Ángel Candela, Belen Ponte, Fernando Liaño, María Laura García-Bermejo

**Affiliations:** 1 miRNAs-based Biomarkers and Therapeutic Targets Unit, Instituto Ramón y Cajal de Investigación Sanitaria (IRYCIS), Carretera de Colmenar, Madrid, Spain; 2 Department of Intensive Medicine, University Hospital Ramón y Cajal, Carretera de Colmenar, Madrid, Spain; 3 Department of Anesthesia and Reanimation, University Hospital Ramón y Cajal, Carretera de Colmenar, Madrid, Spain; 4 Department of Internal Medicine, University Hospital of Geneve, Geneva, Switzerland; 5 Department of Nephrology, University Hospital Ramón y Cajal, Madrid, Spain. Instituto Ramón y Cajal de Investigación Sanitaria (IRYCIS), Carretera de Colmenar, Madrid, Spain; University of Torino, ITALY

## Abstract

In the last decade, Acute Kidney Injury (AKI) diagnosis and therapy have not notably improved probably due to delay in the diagnosis, among other issues. Precocity and accuracy should be critical parameters in novel AKI biomarker discovery. microRNAs are key regulators of cell responses to many stimuli and they can be secreted to the extracellular environment. Therefore, they can be detected in body fluids and are emerging as novel disease biomarkers. We aimed to identify and validate serum miRNAs useful for AKI diagnosis and management. Using qRT-PCR arrays in serum samples, we determined miRNAs differentially expressed between AKI patients and healthy controls. Statistical and target prediction analysis allowed us to identify a panel of 10 serum miRNAs. This set was further validated, by qRT-PCR, in two independent cohorts of patients with relevant morbi-mortality related to AKI: Intensive Care Units (ICU) and Cardiac Surgery (CS). Statistical correlations with patient clinical parameter were performed. Our results demonstrated that the 10 selected miRNAs (miR-101-3p, miR-127-3p, miR-210-3p, miR-126-3p, miR-26b-5p, miR-29a-3p, miR-146a-5p, miR-27a-3p, miR-93-3p and miR-10a-5p) were diagnostic biomarkers of AKI in ICU patients, exhibiting areas under the curve close to 1 in ROC analysis. Outstandingly, serum miRNAs estimated before CS predicted AKI development later on, thus becoming biomarkers to predict AKI predisposition. Moreover, after surgery, the expression of the miRNAs was modulated days before serum creatinine increased, demonstrating early diagnostic value. In summary, we have identified a set of serum miRNAs as AKI biomarkers useful in clinical practice, since they demonstrate early detection and high diagnostic value and they recognize patients at risk.

## Introduction

Acute Kidney Injury (AKI) is a frequent clinical syndrome with incidence of 550 cases per 100,000 individuals, similar to the myocardial infarction rate per year [1, 2]. In hospitalized patients, AKI incidence can reach 13%, depending on the diagnostic criteria employed [3]. In the setting of cardiac surgery, AKI incidence reaches approximately 35% and kidney injury severity is critical for patient outcome after the intervention [4]. AKI still presents a very high morbidity and mortality, especially in intensive care units (ICU), where mortality can reach 50–70%. AKI survivors exhibit a significant risk of developing chronic morbidities, such as chronic kidney disease (CKD), or accelerated end-stage renal disease development.

Despite great advances in prevention strategies, patient classification and technology, no efficient therapy is available once AKI occurs, most probably due to delay in the diagnosis. Even though its widespread use, serum creatinine shows important and well-known limitations for assessing Glomerular Rate Filtration (GFR) [5], including the delay in the diagnosis or their values modification by drugs. Novel biomarkers such as NGAL or Cistatin did not exhibit the diagnostic value expected in all the AKI populations. All these caveats impact on the current AKI definitions used in clinical practice and point out the urgent need for new AKI biomarkers identification in order to improve AKI diagnosis and patient management. In fact, novel biomarkers for structural AKI are emerging since they could lead to a better stratification of patients and provide additional information to the functional ones [6], including miRNAs

miRNAs are small (20–25 nucleotides) endogenous RNA molecules which post-transcriptionally regulate gene expression [7]. miRNAs play critical roles in kidney development, homeostasis and function, including kidney physiology [8]. Additionally, they are also key mediators in renal responses to injury, such as ischemia/reperfusion damage [9, 10]. It has been widely demonstrated that miRNAs can also be secreted to the extracellular environment, with potential functional consequences [11, 12]. This secretion allows them to be detected in a wide range of cell-free body fluids including urine or serum. Indeed, changes in serum miRNA profiles have been proposed as biomarkers for a wide range of diseases such as cancer, cardiovascular disease, and nephropathies, among others [13]. Thus, it is conceivable that serum miRNAs could be a useful tool for AKI diagnosis. In fact, recent reports have identified miRNAs associated with AKI in urine from ICU patients [14] as well as in plasma and in serum of animal experimental models of AKI [15], including toxic AKI [16].

In this pilot study, we have aimed to identify and validate a panel of serum miRNAs that could be useful for AKI diagnosis in clinical practice. We have used serum samples from ICU patients and cardiac surgery (CS) patients, two clinical situations where AKI incidence is high. We also aimed to study whether this set of miRNAs could also provide further clinical insight, thus allowing better monitoring and management of AKI patients.

## Material and Methods

### 1. Human serum sample collection and storage

All our patient studies were approved by the Institutional Review Boards. Written informed consent document was received from participants prior to inclusion in the study. Samples and data from patients as well as healthy volunteers were provided by the University Hospital Ramóxn y Cajal-IRYCIS Biobank, integrated in the Spanish Hospital Biobanks Network (RetBioH; www.redbiobancos.es), with appropriate approval of the Ethical and Scientific Committees and according to the quality standard guidelines (ISO 9001:2008). Serum was obtained by centrifugation at 1,100 g for 10 minutes at 4°C, then aliquoted and immediately stored at -80°C. Stability of miRNAs after freeze/thaw cycles was demonstrated.

### 2. Patient Cohorts

Our study is a Pilot observational prospective study with different patient cohorts used for profiling or validation studies:

#### 2.1. Cohorts for miRNA profiling experiments

*2*.*1*.*1*. Samples from healthy volunteers with normal renal function and no major pathologies were divided into two pools (n = 5 individual each) taking into account age and sex distribution, in order to minimize interpersonal variations and increase the robustness of difference between healthy individuals and patients.

*2*.*1*.*2*. AKI patients: samples of this cohort were selected from Biobank collection (Reference: AD4-FRA). This collection is composed of patients exhibiting AKI subsequent to acute tubular necrosis, exclusively, excluding other pathologies and multiorganic failure.

#### 2.2 Cohorts for miRNA validation in Intensive Care Units (ICU) patients

*2*.*2*.*1*. Samples are from 20 healthy individuals in the Biobank collection (Reference AD5- BCS). These samples are coming from Hospital blood donors and they are used as healthy controls.

*2*.*2*.*2*. Samples from 35 ICU patients diagnosed with AKI in the context of multiorganic failure were obtained from Biobank collection (Reference AD1-FMO). AKI was diagnosed using serum creatinine AKIN criteria [17]. Patients were diagnosed with pre-renal azotemia when serum creatinine returned to baseline after adequate treatment in less than 72 hours. For each patient, we analyzed serum samples collected at day 0 (Diagnosis), day 1, day 2, day 3 and day 7.

#### 2.3. Cohorts for miRNA validation in Cardiac Surgery (CS) Patients

41 CS patients were collected and stored in Ramon y Cajal Hospital Biobank (collection reference AD3-CEC). To be included in this cohort, patients conform to the following criteria:

- **I:** paediatric naïve patients operated with cardiopulmonary bypass (10 individuals).
- **II:** adult patients with 0–2 points in Thakar score system for AKI prediction after surgery [18]. (10 individuals).
- **III:** adult patients with altered baseline renal function and > 5 points in Thakar score (14 individuals).
- **IV:** adult patients with normal renal function and > 5 points in Thakar system (7 individuals).

AKI was diagnosed using AKIN [17] and RIFLE [19] serum creatinine criteria and creatinine kinetics [20] definitions independently. For each patient, serum samples were collected: before surgery (Baseline), immediately post-surgery (IPS), and at 1, 2,3 and 7 days after surgery.

### 3. RNA extraction from serum samples

Total RNA was extracted with miRNeasy mini kit (Qiagen) using an optimized protocol. Briefly, 250 μl aliquots were thawed on ice and centrifuged 1,000g for 5 minutes at 4°C to remove debris. For each sample, 800 μl of phenol master mix was prepared, containing 1 μg of MS2 RNA (Roche). For data normalization during miRNA detection by quantitative real time PCR (RT-qPCR), 2x10^6^ copies of Spike-In synthetic RNA oligo (Unknown sequence; Exiqon) were added to this master mix. 750 μl of master mix were used for each 200 μl of precleared serum sample. The following steps were carried out according to manufacturer´s instructions. RNA was eluted by adding 50 μl of nuclease free water.

### 4. microRNA RT-qPCR Array and data analysis

Circulating miRNA profiling experiments were carried out using Taqman Low Density Arrays for miRNAs (Applied Biosystems). For each sample, total RNA from three different aliquots was extracted as detailed above to avoid aliquot or RNA extraction procedure variability. After RNA quantification and quality checking, RNA samples were pooled for further steps. Reversetranscription was performed using Megaplex RT primer pool panel A and B and Taqman miRNA retrotranscription kit (Applied Biosystems) following manufacturer´s instructions.

PCR determinations were performed using Taqman Array Human microRNA cards (Applied Biosystems) following manufacturer’s instructions. All reactions were carried out in a 7900HT Fast Real-Time PCR System and raw Cq were obtained using SDS v2.3 software (Applied Biosystems).

Raw Cq values were exported and analyzed in Excel (Microsoft). First, miRNAs not expressed in any sample were eliminated. After this step, all values showed as “undetermined” were substituted by 40 as Cq value. Next, ΔCq values were calculated (ΔCq = miRNA Cq—Reference gene Cq) using as reference control the mean of the Cq values of all expressed miRNAs (Cq values <35) [21]. Next, miRNA with inconsistent ΔCq values between control samples (ΔΔCq>1) were also excluded from the study.

After these initial selection steps, based on technical criteria, fold changes were calculated using 2^-ΔΔCq^ method. miRNAs showing statistically significant changes over ±2 fold were selected.

### 5. microRNA quantification by LNA probes

Serum miRNAs were detected and quantified by Real-Time PCR using Universal RT miRNA PCR System (Exiqon). 4 μl of the eluted RNA was used as template for retrotranscription in a final volume of 20 μl. cDNA was diluted 1/11 with nuclease-free sterile water and 4 μl was used as template for PCR reaction.

Real time PCR detection was performed using SYBR Green and specific commercially available probes (Exiqon) for each miRNA of interest. Master Mix preparation and temperature cycles were performed following manufacturer’s instructions. All reactions were carried out in triplicates in Light Cycler 480 equipment (Roche) and Cq values were calculated using 2^nd^ derivative method (Light Cycler 480 Software 1.5, Roche). miRNA expression values are expressed as ΔCq, obtained from the following formula: ΔCq = miRNA Cq—Spike In Cq.

As previously mentioned, miRNA stability after few freeze/thawing cycles was checked in our lab demonstrating high stability, as previously reported [22].

### 6. Statistical Analysis

Normal distribution of variables was assessed with the Saphiro-Wilk test. For data with normal distribution, t-test and ANOVA with post hoc Bonferroni correction for multiple comparisons were used after assessing homogeneity of variances with the Levene test. For group comparison of non-normal distributed data, Kruskal—Wallis test was used. Intergroup differences of non-normal data were assessed with post hoc Mann—Whitney U-tests. Spearman Rho correlation coefficient was used for correlation studies.

ROC curve test was used to evaluate diagnostic performance. P<0.05 was considered significant. Statistical analyses were performed using Statistical Package for the Social Sciences (SPSS) software version 19.0.

## Results

### Identification of serum miRNAs differentially expressed between healthy volunteers and AKI patients

We performed a screening experiment using RT-qPCR arrays, including 786 miRNAs. For this initial experiment, two pools of healthy people (n = 5 each) and 4 AKI patients were included. Clinical settings of these patients are shown in Table 1.

**Table 1:** Clinical settings of AKI patients and healthy control pools included in the screening experiment.

	Age	Gender	AKIN Stage	Serum Creatinine (mg/dl)
**Healthy Control**				
**Pool 1 (n = 5)**	32.8 ± 5.8*	2 Men/3 Women	N/A	0.85 ± 0.12*
**Pool 2 (n = 5)**	36.6 ± 8.6*	3 Men/2 Women	N/A	0.74 ± 0.13*
**AKI Patients**				
**1**	41	Man	3	13.00
**2**	45	Woman	3	4.81
**3**	63	Woman	3	1.53
**4**	47	Man	2	5.01

N/A: Not Applicable

* Data expressed as mean ± standard deviation

After elimination of unexpressed or inconsistently expressed miRNAs, based on technical criteria, miRNAs with more than ±2 folds change in expression and statistical significance were selected (Table 2).

**Table 2:** miRNAs selected from the massive screening experiment to be tested as potential AKI biomarkers.

miRNA ID	Accession Number	Fold Change	p-value
hsa-miR-**101-3p**	MIMAT0000099	-2.75	0.049
hsa-miR-**127-3p**	MIMAT0000446	-2.01	0.047
hsa-miR-**210-3p**	MIMAT0000267	-3.15	0.030
hsa-miR-**126-3p**	MIMAT0000445	-5.75	0.036
hsa-miR-**26b-5p**	MIMAT0000083	-7.52	0.024
hsa-miR-**29a-3p**	MIMAT0000086	-6.62	0.031
hsa-miR-**146a-5p**	MIMAT0000449	-8.50	0.021
hsa-miR-**27a-3p**	MIMAT0000084	-5.09	0.001
hsa-miR-**93-3p**	MIMAT0004509	-22.03	0.017
hsa-miR-**10a-5p**	MIMAT0000253	-32.21	0.001

In order to contextualize the selected miRNAs in AKI, we performed a bioinformatics functional analysis. For each miRNA, potential targets were downloaded from Targetscan Human 5.1 database (http://www.targetscan.org/vert_50/) [23]. Target gene list were then analyzed using the online Bioinformatics Database for Annotation, Visualization and Integrated Discovery (DAVID) (http://david.abcc.ncifcrf.gov/tools.jsp), using default settings [24]. As can be observed in S1 Table, selected miRNAs show targets genes involved in renal response during AKI.

After selection, this panel of miRNAs was validated in a cohort of ICU patients with AKI and a cohort of cardiac surgery patients. Both clinical contexts were chosen based on the high incidence of AKI.

### Serum miRNAs are diagnostic Biomarkers of AKI in ICU Patients

**Fig 1:**
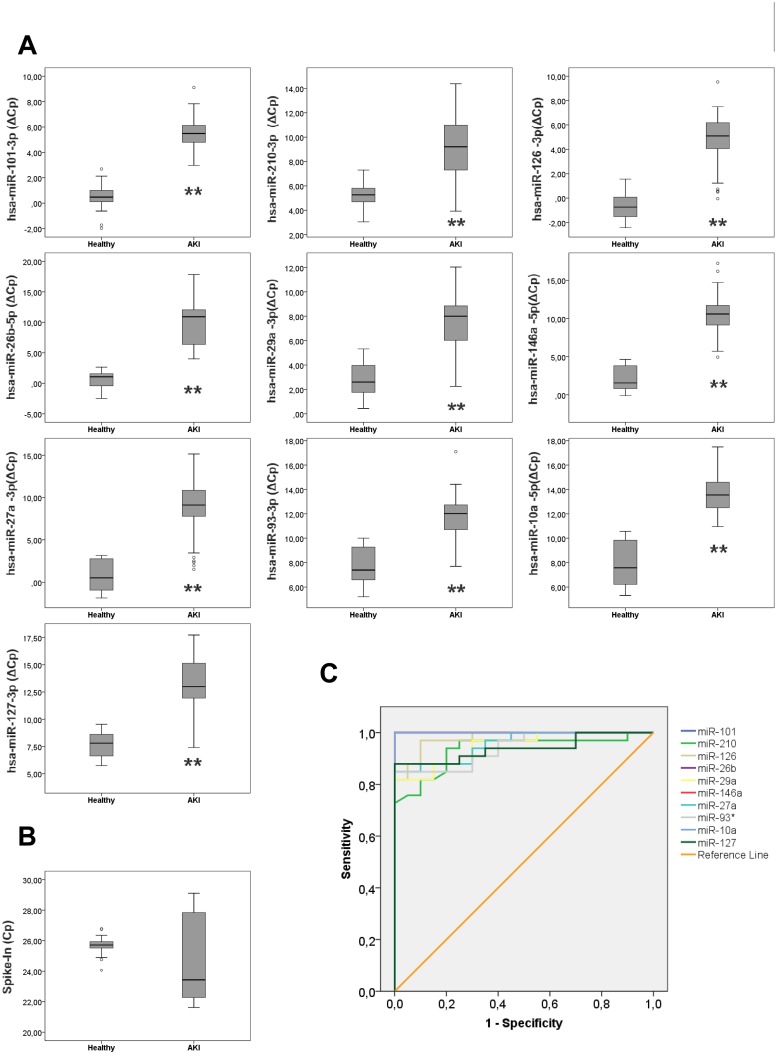
Serum microRNAs are diagnostic biomarkers of AKI in ICU patients. (A) miRNAs were detected by RT-qPCR in serum samples from ICU patients and healthy controls. miRNA levels are expressed as ΔCp and data are presented as median and interquartile range. Asterisks indicate statistical significance (*P<0.05; ** P<0.01) (B) Spike-In raw Cp values as control. (C) ROC curve analysis.

Clinical settings for ICU patients and controls used for validation can be found in Table 3. Selected serum microRNAs were detected in samples obtained at the time of diagnosis (Fig 1A). In agreement with data obtained in the screening experiment, studied miRNAs were markedly downregulated (higher ΔCp values) in AKI samples compared to healthy controls. Fig 1B shows raw Cp values of Spike-In RNA in these samples, used as control for miRNA quantification. These values show no statistical significance between healthy and AKI samples (p-value = 0.081), demonstrating its reliability as RT-qPCR reference gene control.

**Table 3:** Clinical settings of ICU patient cohort and healthy controls.

	Healthy Control (n = 20)	AKI Patients (n = 35)
Age (years)	38.35 ± 11.42	64.9 ± 15.5
Male (n, %)	12, 60%	28, 80%
Female (n, %)	8, 40%	7, 20%
**Serum Creatinine** (mg/dl)		
	0.76 ± 0.16	2.73 ± 4.47
**AKIN Grade** (Patient number)		
1	N/A	11
2	N/A	9
3	N/A	15
**AKI Origin**		
Pre-renal	N/A	16
ATN	N/A	19
**Prognostic Score**		
SOFA	N/A	6.37 ± 3.68

N/A: Not Applicable

Receiver Operator Characteristic (ROC) curve analysis was performed to study the diagnostic performance of each of these miRNAs for AKI. As can be observed in Fig 1C and Table 4, all miRNAs presented areas under the curve (AUC) values between 0.9 and 1. Additional information regarding ROC analysis is shown in S2 Table.

**Table 4:** ROC Curve analysis of ICU patients in the moment of AKI diagnosis compared to healthy control.

microRNA	ROC Analysis Day 0 (Diagnosis)		
	AUC	S.E.M.	p-value
**miR-101-3p**	*1*.*000*	*0*.*000*	*0*.*000*
**miR-210-3p**	*0*.*935*	*0*.*034*	*0*.*000*
**miR-126-3p**	*0*.*979*	*0*.*015*	*0*.*000*
**miR-26b-5p**	*1*.*000*	*0*.*000*	*0*.*000*
**miR-29a-3p**	*0*.*948*	*0*.*27*	*0*.*000*
**miR-146a-5p**	*1*.*000*	*0*.*000*	*0*.*000*
**miR-27a-3p**	*0*.*955*	*0*.*025*	*0*.*000*
**miR-93-3p**	*0*.*942*	*0*.*029*	*0*.*000*
**miR-10a-5p**	*1*.*000*	*0*.*000*	*0*.*000*
**miR-127-3p**	*0*.*939*	*0*.*033*	*0*.*000*

S.E.M: Standard error of the mean

**Fig 2:**
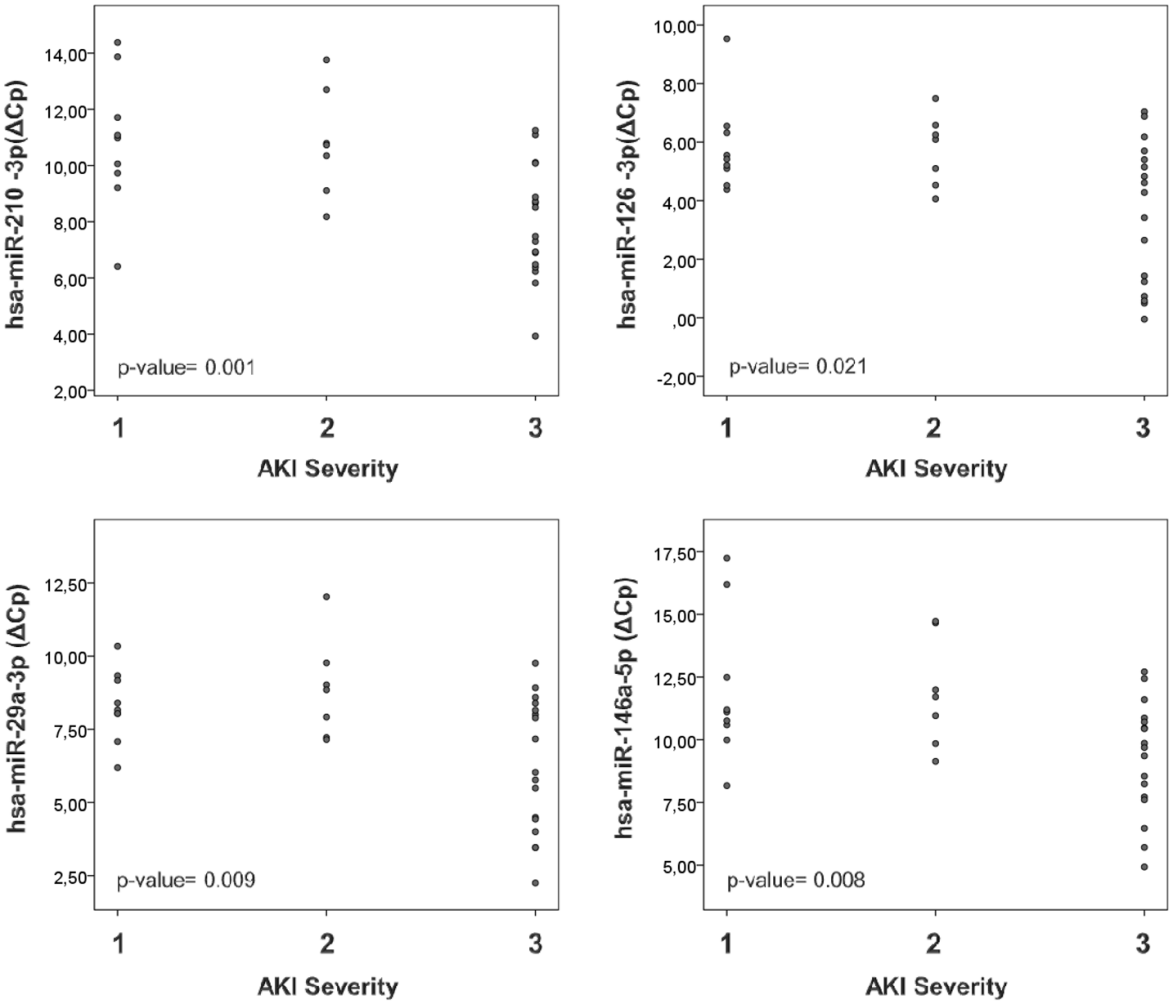
Serum microRNAs indicate AKI severity. Serum miRNAs were detected by qRT-PCR in samples from ICU patients at the moment of AKI diagnosis. (A) Serum levels of miR-210-3p, miR-126-3p, miR-29a-3p and miR-146a-5p correlate with AKI severity determined by AKIN classification and with increasing severity from 1 to 3 stage. miRNA expression levels are shown as ΔCp values and each patient is represented by an individual dot. Spearman correlation was performed for each miRNA and p-values are shown in the corresponding plots. Asterisks indicate statistical significance (*P<0.05 and ** P<0.01).

Moreover, correlation analysis demonstrated that serum levels of miR-210-3p, miR-126-3p, miR-29a-3p and miR-146a-5p correlate with AKI severity, estimated by AKIN criteria [17] (miR-210-3p p-value = 0.001; miR-126-3p p-value = 0.021; miR-29a-3p p-value = 0.009; miR146a-5p p-value = 0.008) (Fig 2).

These data demonstrate that our panel of serum miRNAs could be used for AKI diagnosis with nearly 100% sensitivity and specificity in this cohort. Additionally, serum levels of miR-210-3p, miR-126-3p, miR-29a-3p and miR-146a-5p could indicate AKI severity in a single determination.

### Serum miRNAs are early Biomarkers of AKI in CS patients

**Fig 3:**
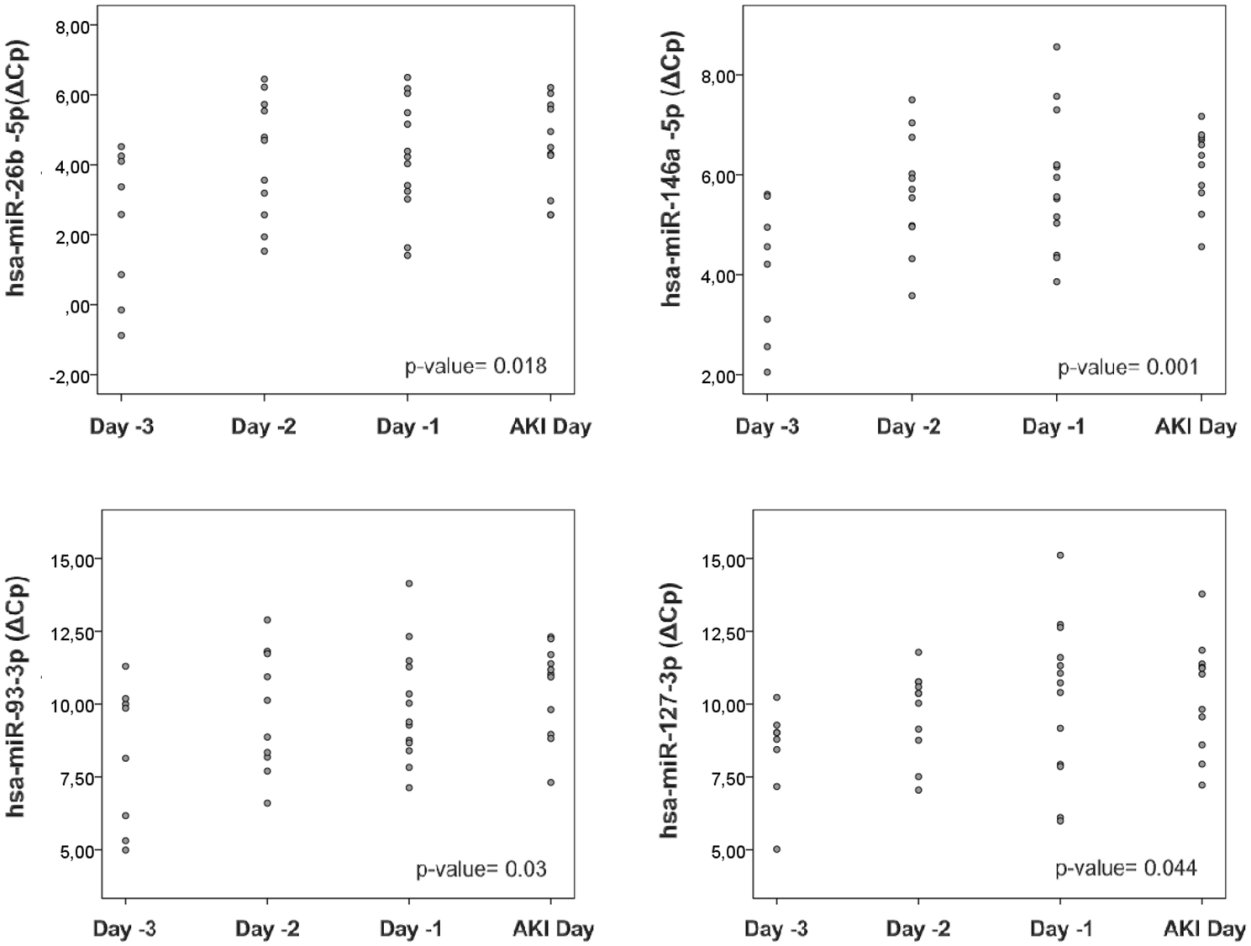
Serum miRNAs are AKI precocious biomarkers in cardiac surgery patients. miR-26b-5p, miR-146a-5p, miR-93-3p and miR-127-3p serum levels are expressed as ΔCp values and data is presented as individual dots. AKI was diagnosed based on AKIN serum creatinine criteria. Spearman Rho correlation coefficient was calculated and p-values are indicated in each panel.

In addition to the ICU cohort, we have used CS patient samples since this experimental design allow us to monitor and collect samples during AKI development. Clinical settings for these patients can be found in Table 5. We analyzed the expression of our panel of miRNAs in the samples collected before AKI diagnosis (days 1, 2, and 3 before AKI development). Spearman Rho correlation coefficient was calculated to find out statistically significant correlations between miRNA expression and time-points before AKI diagnosis, based on AKIN criteria [17]. As can be observed in Fig 3, miR-26b-5p, miR-146a-5p, miR-93-3p and miR-127-3p showed a progressive downregulation during the days prior to AKI diagnosis.

**Table 5:** Clinical settings of cardiac surgery patient cohort.

	I(n = 10)	II(n = 10)	III(n = 14)	IV(n = 7)
Age (years)	52.3 (10.6–108.4)*	65.6 ± 10.4	74.8 ± 6.9	62.0 ± 12.3
Gender (Men/Women)	7/3	5/5	4/10	5/2
BMI (kg/m^2^)	14.5 ± 2.3	27.5 ±3.6	26.7 ± 3.7	28.1 ± 2.7
CPB Time (min)	58.1 ± 20.3	96.6 ± 31.7	101.7 ± 34.7	105.2 ± 33.6
ICU Stay (days)	3 (3–5); 6.9 ± 11.6	3 (2–6);3.6 ± 2.1	6.5 (2–10); 10.3 ± 16.5	7 (2–12.5); 7.3 ± 5.1
Basal Serum Cr. (mg/dl)	0.57 ± 0.1	0.9 ± 0.1	1.2 ± 0.3	0.95 ± 0.3
24h Serum Cr. (mg/dl)	0.55 ± 0.1	0.8 ± 0.2	1.1 ± 0.4	1.02 ± 0.6
Discharge Serum Cr (mg/dl)	0.5 ± 0.1	0.8 ± 0.2	1.19 ± 0.4	1.19 ± 0.6
**Mortality Prediction**				
LogEuroSCORE	N/A	4.08 ± 3.05	9.7 ± 4.4	9.8 ± 7.8
Cleveland Score	N/A	1.8 ± 0.9	3.83 ± 1.0	4.25 ± 1.0
SRI Score	N/A	1 ± 0.5	3.45 ± 0.5	3.0 ± 0.5
**AKI Development**				
RIFLE Cr (n)	1	1	4	4
RIFLE Diuresis (n)	0	4	3	1
Estimated GFR (n)	0	3	4	4
AKIN Cr. (n)	1	4	9	4
Cr. Kinetics (n)	1	1	7	1

BMI: Body Mass Index

N/A: Not Applicable

LOS: Length of Stay.

Cr: Creatinine.

* Age for children (median, 25^th^— 75^th^ percentiles) is expressed in months

These data, in agreement with the data obtained in ICU patients, indicate that serum miRNAs are diagnostic biomarkers of AKI. Moreover, miRNAs were able to predict AKI development several days before serum creatinine, allowing monitoring and serving as early AKI biomarkers.

### Serum miRNAs are Biomarkers of AKI Predisposition in CS

In order to identify AKI predisposition biomarkers in CS population, miRNAs included in our panel were determined in basal serum samples of CS patients, which were obtained before the intervention. Those miRNAs showing significant changes using all AKI definitions were considered as real biomarkers of AKI development after surgery.

**Fig 4:**
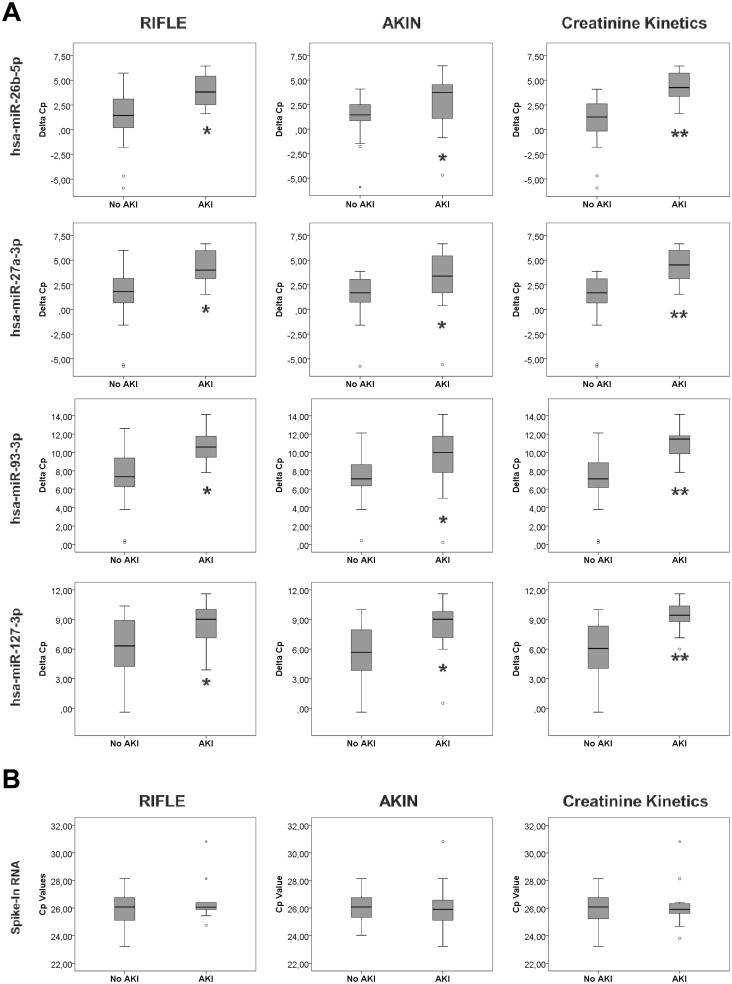
Serum miRNAs estimated before cardiac surgery identify patients in risk of AKI development. (A) miRNAs were detected by RT-qPCR in serum samples obtained before surgery, expressed as ΔCp. Data are presented as median and interquartile range. Asterisks indicate statistical significance (*P<0.05; ** P<0.01). (B) Spike-In raw Cp values are shown as control.

miR-26b-5p, miR-27a-3p, miR-93-3p and miR-127-3p were significantly downregulated in basal serum samples of those patients who develop AKI after surgery, compared to those who do not develop AKI (Fig 4A), indicating that these miRNAs could be biomarkers of AKI predisposition in this cohort. Spike-In raw Cp values do not significantly change between patients who develop AKI and those who do not develop AKI after surgery in any of the used classifications (RIFLE: p-value = 0.450; AKIN: p-value = 0.725; Cr Kinetics: p-value = 1) (Fig 4B).

**Fig 5:**
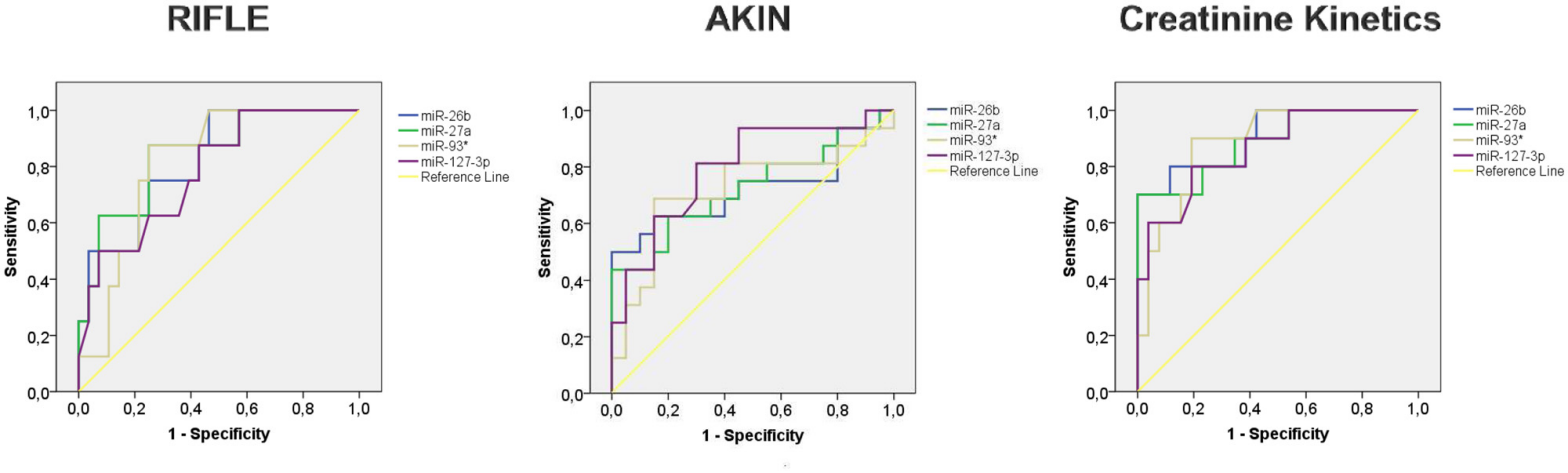
ROC analysis of serum miRNAs as AKI risk biomarkers. ROC curves of miR-26b-5p, miR-27a-3p, miR-93-3p and miR-127-3p expression levels in serum, determined by qRT-PCR, in samples obtained before surgery.

As it is shown in Fig 5 and Table 6, these miRNAs exhibited significant AUC values between 0.7–0.9, depending on the AKI definition criteria used. Additional information regarding ROC analysis is shown in S3 Table.

**Table 6:** ROC Curve analysis of serum miRNAs estimated in samples obtained before surgery.

	RIFLE		AKIN		Creatinine Kinetics	
	AUC	p-value	AUC	p-value	AUC	p-value
**miR-26b-5p**	*0*.*821*	*0*.*006*	*0*.*722*	*0*.*024*	*0*.*908*	*0*.*000*
**miR-27a-3p**	*0*.*844*	*0*.*007*	*0*.*725*	*0*.*022*	*0*.*888*	*0*.*000*
**miR-93-3p**	*0*.*815*	*0*.*007*	*0*.*719*	*0*.*026*	*0*.*887*	*0*.*000*
**miR-127-3p**	*0*.*783*	*0*.*016*	*0*.*795*	*0*.*003*	*0*.*863*	*0*.*001*

These results strongly suggest that these four miRNAs, determined before surgery, could be used for identification of patients at risk of AKI development.

## Discussion

In this pilot study, we identified and validated a set of miRNAs, including miR-101-3p, miR-127-3p, miR-210-3p, miR-126-3p, miR-26b-5p, miR-29a-3p, miR-146a-5p, miR-27a-3p, miR-93-3p and miR-10a-5p, as potential biomarkers of AKI in ICU and CS patients.

Genome wide profiling experiments have demonstrated that some of these miRNAs (miR-10a, miR-27a, miR-29a, miR-101 and miR-210) are highly expressed in human kidney tissue [25, 26]. Indeed, it has been confirmed the presence of some of these miRNAs in serum microvesicles [27].

Some of these miRNAs have also been related to kidney injury. miR-127 has been recently demonstrated as a regulator of proximal tubule cell adhesion and trafficking during Ischemia/Reperfusion (I/R) injury [9]. miR-146a is modulated in an experimental model of renal I/R in mice [28] and in patients with IgA nephropathy, where its levels in renal tissue and urine were correlated with injury severity [29]. miR-29a is associated with kidney fibrosis regulation and CKD progression. This miRNA is regulated by Transforming growth Factor β (TGF-β) and its expression prevents fibrosis by avoiding extracellular matrix deposition in renal parenchyma [30].

Remarkably, Lorenzen JM. et al., reported that miR-210-3p levels in plasma predicted survival in critically ill patients with AKI. In this study, AKI patients presented higher levels of miR-210-3p compared to healthy controls [31]. Additionally, a urine miRNA profile for AKI has been recently published [32]. The data presented in these reports do not match with our serum miRNA profile and levels, most probably due to the use of plasma and urine instead of serum. miRNAs secreted to different body fluids and blood fractions could have different biological significance and functions.

Our data demonstrate that our panel of miRNAs shows a high diagnostic value with AUC values between 0.9 and 1 in ROC analysis. This discriminative power is higher than that reported for other AKI biomarkers currently in development, including NGAL, Cystatin C or IL-18 [33].

An important characteristic of an AKI biomarker is the ability to achieve a precise quantification of renal injury. Our results demonstrate that serum levels of miR-210-3p, miR-126-3p, miR-29a-3p and miR-146a-5p show a significant correlation with AKIN stage classification, indicating that these miRNAs could be used for deciphering AKI severity. Among the wide range of new AKI biomarkers, only serum Cystatine-C levels correlate with AKI severity [34], although others such as plasma NGAL can predict AKI progression in severity [35].

On the other hand, precocious AKI diagnosis is another valuable characteristic of the new generation of AKI biomarkers. To assess this issue, we studied the expression of our panel of miRNAs in a cohort of patients which underwent CS. This procedure can be considered a quasi-experimental design since the moment of kidney insult is known and AKI development and outcome can be closely monitored. Several biomarkers have been tested in this clinical context to predict AKI immediately or few hours after intervention. They have demonstrated very high predictive values in pediatric populations but more variable results in adults. NGAL presented AUC values ranging from 0.54 to 0.87, IL-18 from 0.55 to 0.87, Cystatin-C from 0.73 to 0.76 and from 0.68 to 0.78 in the case of KIM-1 [36, 37].

In this regard, our correlation studies demonstrate that miR-26b-5p, miR-146a-5p, miR-93-3p and miR-127-3p progressively decrease their expression levels in serum during the days prior to AKI diagnosis by serum creatinine. These data demonstrate that serum miRNAs can detect AKI development several days before serum creatinine. It is important to emphasize that miR-146a-5p, which demonstrated a very high diagnostic value in ICU patients, presents here a strong and significant downregulation during previous days to AKI. These data confirm that this miRNA could be considered a precise and early AKI diagnostic tool in several clinical contexts.

Remarkably, basal levels of miR-26b-5p, miR-27a-3p, miR-93-3p and miR-127-3p, measured before surgery, can robustly predict AKI development after CS, independently of the AKI criteria used for diagnosis. They show a high predictive value with AUC values between 0.7 and 0.9. This evidence demonstrates that several miRNAs included in our panel can be considered as markers of AKI predisposition. Only KIM-1 has demonstrated this ability by predicting stage 3 AKI after CPB with an AUC value of 0.8 [38]. In fact, our data demonstrate for the first time that serum miRNAs can predict AKI before surgery independently of its severity. This valuable information could have a great impact in clinical practice, since it would allow the detection of patients at risk of AKI development and would permit the application of prevention strategies. None of the other biomarkers available so far can provide such benefit.

## Conclusions

In this pilot study, we have identified and validated a panel of serum microRNAs as novel biomarkers of AKI in ICU and CS patients. These serum miRNAs present a high diagnostic value in ICU patients and their expression levels correlated with AKI severity.

Besides, in patients submitted to CS, serum miRNA levels could identify populations at risk for AKI development after surgery. This ability has not been reported for any AKI biomarkers up to this moment. Moreover, serum levels of these miRNAs progressively decreased days before AKI diagnosis, pointing out their potential role as early injury biomarkers.

Based on these results and consistently with other reports, serum miRNAs could be considered as a new generation of AKI diagnostic biomarkers, helpful for improvement of AKI patient management in clinical practice.

## Supporting Information

S1 Table DAVID functional classification of the putative targets of the selected miRNAs. Functional categories showed in this table, clustering miRNAs targets, are extracted from KEGG and GO databases.
(DOCX)

S2 Table Statistical features of ROC analysis for miRNAs definition as AKI diagnostic biomarkers in ICU patients. (DOCX)

S3 Table Statistical features of ROC analysis for miRNAs definition as AKI predisposition biomarkers in CS patient samples. (DOCX)
