# Supplementary material for: A Pilot Study Identifying a Set of microRNAs As Precise Diagnostic Biomarkers of Acute Kidney Injury
Source: PLoS One. 2015 Jun 16;10(6):e0127175. doi: 10.1371/journal.pone.0127175 (PMC4469584; doi:10.1371/journal.pone.0127175)
Supplement: S1 Table — Functional categories showed in this table, clustering miRNAs targets, are extracted from KEGG and GO databases. (DOCX) [file pone.0127175.s001.docx]

**Supplementary Table 1: DAVID functional classification of the putative targets of the selected miRNAs.** Functional categories showed in this table, clustering miRNAs targets, are extracted from KEGG and GO databases.

| **microRNA** | **Functional Category** |
| --- | --- |
| **hsa-miR-101-1** | Kidney development |
|  | Cell adhesion |
|  | Endocytosis |
| **hsa-miR-127-3p** | Ionic channel |
|  | Cell adhesion molecules |
|  | Epithelium development |
| **hsa-miR-210** | Basement membrane |
|  | Actin cytoskeleton organization |
|  | Tube development |
| **hsa-miR-126** | Kidney development |
|  | Microtubule-based transport |
|  | Cell-cell adherent junction |
| **hsa-miR-26b** | Epithelial tube morphogenesis |
|  | ATP binding |
|  | Cytoskeleton |
| **hsa-miR-29a** | Extracellular matrix |
|  | Focal adhesion |
|  | Collagen type IV |
| **hsa-miR-146a** | Microtubule cytoskeleton |
|  | Intracellular transport |
|  | Kidney development |
| **hsa-miR-27a** | Kidney development |
|  | Endocytic activity |
|  | Epithelial tube morphogenesis |
| **hsa-miR-93*** | Endocytosis |
|  | GTPase regulator activity |
|  | Tube development |
| **hsa-miR-10a** | Endocytosis |
|  | Adherent junction |
|  | Focal adhesion |

*hsa: Homo sapiens*
