# Supplementary material for: A Pilot Study Identifying a Set of microRNAs As Precise Diagnostic Biomarkers of Acute Kidney Injury
Source: PLoS One. 2015 Jun 16;10(6):e0127175. doi: 10.1371/journal.pone.0127175 (PMC4469584; doi:10.1371/journal.pone.0127175)
Supplement: S2 Table — (DOCX) [file pone.0127175.s002.docx]

**Table S2:** Statistical features of ROC analysis for miRNAs definition as AKI diagnostic biomarkers in ICU patients .

| **microRNA** | **ROC Analysis Day 0 (Diagnosis)** | | | **95% confidence Interval** | |
| --- | --- | --- | --- | --- | --- |
|  | **AUC** | **S.E.M.** | **p-value** | **Lower Limit** | **Upper Limit** |
| **miR-101-3p** | *1.000* | *0.000* | *0.000* | *1.000* | *1.000* |
| **miR-210-3p** | *0.935* | *0.034* | *0.000* | *0.868* | *1.002* |
| **miR-126-3p** | *0.979* | *0.015* | *0.000* | *0.949* | *1.009* |
| **miR-26b-5p** | *1.000* | *0.000* | *0.000* | *1.000* | *1.000* |
| **miR-29a-3p** | *0.948* | *0.27* | *0.000* | *0.896* | *1.001* |
| **miR-146a-5p** | *1.000* | *0.000* | *0.000* | *1.000* | *1.000* |
| **miR-27a-3p** | *0.955* | *0.025* | *0.000* | *0.906* | *1.003* |
| **miR-93-3p** | *0.942* | *0.029* | *0.000* | *0.885* | *1.000* |
| **miR-10a-5p** | *1.000* | *0.000* | *0.000* | *1.000* | *1.000* |
| **miR-127-3p** | *0.939* | *0.033* | *0.000* | *0.876* | *1.004* |

*S.E.M: Standard error of the mean*
