# Supplementary material for: A Pilot Study Identifying a Set of microRNAs As Precise Diagnostic Biomarkers of Acute Kidney Injury
Source: PLoS One. 2015 Jun 16;10(6):e0127175. doi: 10.1371/journal.pone.0127175 (PMC4469584; doi:10.1371/journal.pone.0127175)
Supplement: S3 Table — (DOCX) [file pone.0127175.s003.docx]

**Table S3:** Statistical features of ROC analysis for miRNAs definition as AKI predisposition biomarkers in CS patient samples.

| **RIFLE Criteria** | | | | | |
| --- | --- | --- | --- | --- | --- |
| **microRNA** | **ROC Analysis Day 0 (Diagnosis)** | | | **95% confidence Interval** | |
|  | **AUC** | **S.E.M.** | **p-value** | **Lower Limit** | **Upper Limit** |
| **miR-26b-5p** | *0.821* | *0.08* | *0.006* | *0.69* | *0.095* |
| **miR-27a-3p** | *0.844* | *0.08* | *0.007* | *0.72* | *0.97* |
| **miR-93-3p** | *0.815* | *0.07* | *0.007* | *0.70* | *0.93* |
| **miR-127-3p** | *0.783* | *0.09* | *0.016* | *0.64* | *0.93* |

| **AKIN Criteria** | | | | | |
| --- | --- | --- | --- | --- | --- |
| **microRNA** | **ROC Analysis Day 0 (Diagnosis)** | | | **95% confidence Interval** | |
|  | **AUC** | **S.E.M.** | **p-value** | **Lower Limit** | **Upper Limit** |
| **miR-26b-5p** | *0.722* | *0.09* | *0.024* | *0.57* | *0.88* |
| **miR-27a-3p** | *0.725* | *0.09* | *0.022* | *0.58* | *0.87* |
| **miR-93-3p** | *0.719* | *0.09* | *0.026* | *0.57* | *0.87* |
| **miR-127-3p** | *0.795* | *0.08* | *0.003* | *0.67* | *0.92* |

| **Creatinine Kinetics Criteria** | | | | | |
| --- | --- | --- | --- | --- | --- |
| **microRNA** | **ROC Analysis Day 0 (Diagnosis)** | | | **95% confidence Interval** | |
|  | **AUC** | **S.E.M.** | **p-value** | **Lower Limit** | **Upper Limit** |
| **miR-26b-5p** | *0.908* | *0.06* | *0.000* | *0.81* | *1.00* |
| **miR-27a-3p** | *0.888* | *0.06* | *0.000* | *0.78* | *0.99* |
| **miR-93-3p** | *0.887* | *0.06* | *0.000* | *0.79* | *0.98* |
| **miR-127-3p** | *0.863* | *0.07* | *0.001* | *0.75* | *0.97* |

*S.E.M: Standard error of the mean*
